# Foetal aortic flow velocity waveforms in healthy and hypertensive pregnant women

**DOI:** 10.1186/1476-7120-12-1

**Published:** 2014-01-27

**Authors:** Luís Guedes-Martins, Ana Cunha, Joaquim Saraiva, Ana Rita-Gaio, Ana S Cerdeira, Filipe Macedo, Henrique Almeida

**Affiliations:** 1 Department of Experimental Biology, Faculty of Medicine, University of Porto, 4200-319 Porto, Portugal; 2 Hospital Centre of Porto EPE, Department of Women and Reproductive Medicine, Largo Prof. Abel Salazar, 4099-001 Porto, Portugal; 3 Obstetrics-Gynecology, Private Hospital Trofa, 4785-409 Trofa, Portugal; 4 Department of Mathematics, Faculty of Sciences, University of Porto, Porto, Portugal; 5 CMUP-Centre of Mathematics, University of Porto, Porto, Portugal; 6 Department of Medicine, Beth Israel Deaconess Medical Center, Harvard Medical School, Boston, MA 02215 USA; 7 Gulbenkian Program for Advanced Medical Education, 1067-001 Lisbon, Portugal; 8 Department of Medicine, Faculty of Medicine, University of Porto, 4200-319 Porto, Portugal; 9 Centro Hospitalar S. João, 4200-319 Porto, Portugal; 10 Obstetrics-Gynecology, Hospital-CUF Porto, 4100 180 Porto, Portugal

**Keywords:** Doppler ultrasonography, Resistance index, Pulsatility index, Foetal aortic artery, Hypertension

## Abstract

**Background:**

The foetal aortic Doppler frequency spectrum is influenced by cardiac output and contractility of the foetal heart as well as vascular compliance, blood viscosity and impedance of the arterial vascular system. The present study aimed at comparing Doppler flow pulsatility (PI) and resistance (RI) indexes of foetal proximal descending aorta (AoF) in the first, second and third trimesters of pregnancy, in low risk women and in those with chronic arterial hypertension, who had normal pregnancy outcomes.

**Methods:**

A longitudinal and prospective study was carried out in 101 singleton pregnancies (71 low-risk pregnancies and 30 with essential hypertension). Multivariate regression had to be considered due to the experiment’s nature: two different indexes were read on the same set of individuals, once at each trimester of the pregnancy [1^st^ (11–14 weeks), 2^nd^ (19–22 weeks) and 3^rd^ (28–32 weeks) trimesters]. The response variable was denoted as index d, in a subject with hypertensive status h (hypertensive or normotensive), at continuous time t.

**Results:**

In both groups, AoF-PI and AoF-RI showed a small, but significant increase from the first to the second (1.850 ± 0.339 *vs* 2.110 ± 0.242 for PI, and 0.829 ± 0.068 *vs* 0.857 ± 0.038 for RI; p < 0.001) and the first to the third (1.850 ± 0.339 *vs* 2.163 ± 0.282 for PI, and 0.829 ± 0.068 *vs* 0.864 ± 0.037 for RI; p < 0.001) trimesters of pregnancy. The global model showed that while AoF-RI trends were converging as time progressed, the AoF-PI values exhibited a divergent trend (p < 0.05).

**Conclusions:**

Chronic stable hypertension in pregnancies with normal outcome, evidences an upward regular trend of foetal descending aorta pulsatility index that is similar to the normotensive condition.

## Introduction

Doppler ultrasound modalities have long been used for evaluation of the cardiovascular system [1,2]. Their application in obstetrics to assess foetal and maternal circulations, namely through analysis of blood flow dynamics and the estimation of impedance, made of Doppler ultrasound an established technique for screening and clinical management of high-risk pregnancies [1,3]. Particular emphasis has been given to the pelvic/uteroplacental circulation study as a mean to identify adverse pregnancy outcomes [4-6]. Starting at early stages of pregnancy [2,6,7], changes in uterine flow in first and second trimesters were found to correlate with subsequent development of hypertensive disorders and intra-uterine growth restriction [8].

Longitudinal studies evaluating both maternal and foetal haemodynamics in uncomplicated pregnancies are of utmost importance to provide data on the normal relationship between maternal and foetal circulations, to allow earlier detection of changes and better understanding of their pathophysiology and risk factors.

A substantial research has focused on the Doppler flow pattern of the foetal aorta, viewed as an indicator of fetoplacental haemodynamics and provider of useful information on global foetal cardiocirculatory dynamics and wellbeing [9,10]. In fact, it has been shown to correlate with the severity of foetal anaemia [11], foetal acid–base status [12], foetal hypoxia [13] and intrauterine growth retardation [3,14,15]. Moreover, the foetal descending aorta is a vessel of large calibre, easily visualized by Doppler ultrasound since the early stages of foetal life, particularly from late first trimester onwards [16]. Its Resistance Index (RI) and Pulsatility Index (PI) provide a good insight into resistance to blood flow and vessel haemodynamics [3,17,18].

Chronic arterial hypertension, a highly prevalent disease in the population, has important implications in pregnancy due to its association with adverse outcomes. Its severity and the presence of end-organ damage impinge on the risk of superimposed preeclampsia, abruptio placentae, foetal growth restriction [19], premature birth and neonatal death [20].

However, in a substantial number of pregnant hypertensive women, the pregnancy outcome is similar to normotensive women. It has been pointed out that this aspect reflects foetal circulatory independence from the mother that enables the induction of adaptive responses to cope with the maternal haemodynamic compromise [21-24].

We hypothesized that such responses may be assessed in major foetal vessels employing Doppler ultrasound, when different maternal circulatory conditions still result in similar normal foetal outcome. Therefore, we aimed at comparing impedance indexes of proximal descending foetal aorta in normotensive and chronic hypertensive women along the pregnancy.

## Patients and methods

### Subjects

The study was approved by the ethics committee of Centro Hospitalar do Porto and all subjects provided informed consent [N/ REF.ª 133/10(086-DEFI/126-CES].

Patients were referred to the hospital by their family doctor according to local pregnancy health policies. We studied singleton pregnancies in healthy women or in women with chronic arterial hypertension without known target organ involvement. Women taking folic acid, vitamin or iron supplements were included. Additionally, acetylsalicylic acid, 100 mg per day, was specifically prescribed to all hypertensive women on their first appointment. Women taking other medications were excluded.

In the first appointment, that coincided with the first ultrasound evaluation, they were observed by a senior specialist who reviewed the patient’s history, verified the absence of diabetes and other endocrine disorders, immune disease, renal and structural heart disease, haematological conditions and chronic infections, and measured blood pressure; gestational age (GA) was verified by sonography to be between 11 and 14 weeks. Hypertension (HT) was defined as systolic pressure ≥140 mmHg and/or diastolic pressure ≥90 mmHg, present before pregnancy or the 20th week [19,25-27]. The average of two measurements taken after a 4 hours period of rest was calculated.

Women that met the inclusion criteria and agreed to participate in the study were then enrolled in a longitudinal prospective study that included a trimestral ultrasound evaluation and the recommended regular blood tests. Body mass index was determined upon biometrical data collected before the ultrasound evaluation.

Pregnancies were closely monitored for the appearance of abnormal conditions. These included foetal abnormal Doppler indexes in the umbilical and middle cerebral arteries, and foetal growth <10th and >90th percentile growth curves [28,29], which were a reason for quitting the follow-up.

All deliveries took place at Centro Hospitalar do Porto and all infants were evaluated by a neonatologist.

### Clinical data and Doppler flow study

**Figure 1:**
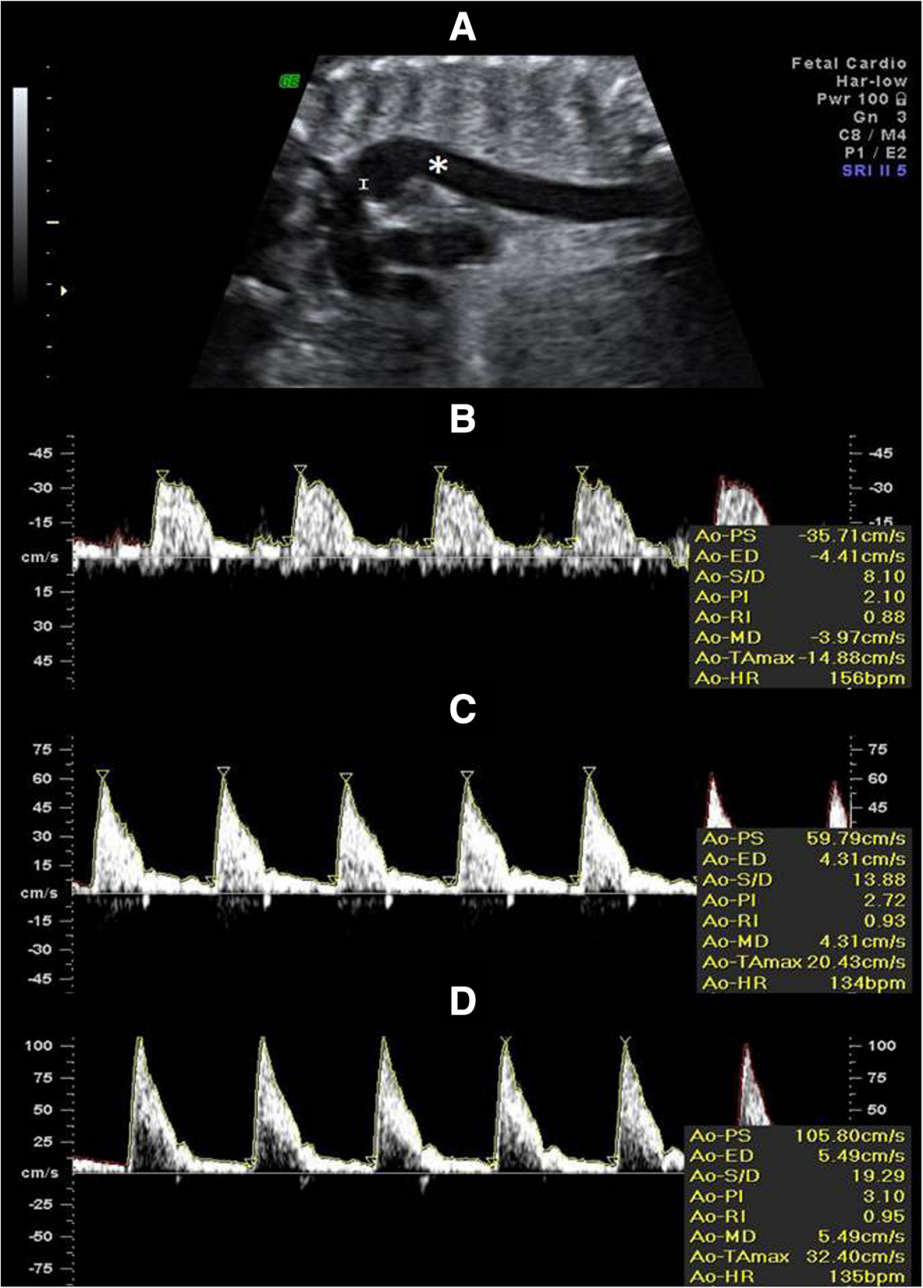
**Ultrasound representative images of the foetal aorta. (A)**: Aortic arch showing the position of the aortic isthmus (I) and the proximal descending aorta (*). **(B)**, **(C)** and **(D)**: Doppler flow waveforms at the first **(B)**, second **(C)** and third **(D)** trimesters, respectively. The flow profile in the foetal aorta is characterized by a steep systolic rise, a postsystolic notch and a low end-diastolic forward flow velocities. The acceleration time is a reflection of cardiac contractility, while the subsequent diastolic phase reflects peripheral vascular impedance. The postsystolic notch develops as a result of aortic valve closure during early ventricular diastole resulting in a slight decrease in flow velocity.

Doppler flow study of foetal proximal descending aorta (AoF) was performed in the 101 women included in the study, immediately before performing the regular obstetrical ultrasound evaluation at the 1^st^ (11–14 weeks), 2^nd^ (19–22 weeks) and 3^rd^ (28–32 weeks) trimesters. AoF-RI and PI Doppler examinations were made using a Voluson E8 or a Voluson 730 Pro (GE Healthcare Technologies, USA) ultrasound machine, equipped with multifrequency transvaginal and transabdominal transducers and all measurements were performed by an investigator with extensive experience in Doppler ultrasound, in order to avoid inter-observer variability. Ultrasound colour Doppler cineloops of the foetal descending aorta were obtained using a C5-2 curved-array transducer, with center frequency of 5 MHz for grayscale imaging and 2.5 MHz for colour Doppler imaging. The colour Doppler maximum velocity setting was adjusted to high velocities so that blood flows of the great vessels were homogeneous in colour, showing no aliasing. The size of the sample volume was adapted to the vessel diameter in order to cover it entirely. All recordings used for measurements were performed in the absence of foetal movements. The scanning plane was adjusted to obtain an insonation angle <30°. All AoF Doppler measurements were sampled and the signal updated until three similar consecutive waveforms were obtained – Figure 1. Pulsatility index (PI) was used as a measure of impedance of the flow of blood distal to the sampling point and automatically calculated according to the formula $\mathrm{PI}=\frac{\left(s-d\right)}{\mathrm{mean}}$ where *s* is the peak *d* is the minimum and the average is the *mean* maximum Doppler shift frequency over the cardiac cycle. Resistance index (RI) was automatically calculated using the formula $\mathrm{RI}=\frac{\left(s-d\right)}{s}$. Smokers were required to abstain from smoking for at least 2 h prior to examination.

AoF assessment is rather simple albeit on occasions, particularly the first trimester of pregnancy, its visualization was sometimes difficult, consequent to frequent dorsoanterior position and ultrasound attenuation caused by the spine. To overcome this difficulty, in the current study, foetal stimulation with abdominal probe and maternal mobilization into lateral decubitus was successfully employed, in agreement with other’s observations [3,18].

### Statistical analysis

Univariate data analysis comprised appropriate statistical methods: chi-square test or Fisher’s test (as adequate) for the study of independence amongst two factors, t-test for the difference of means in two independent populations, and one-way analysis of variance for repeated measurements with Tukey’s multiple comparisons test for the assessment of significant statistical differences across more than two means in paired populations.

Multiple linear regression models with errors, that were allowed to be correlated and/or to have unequal variances, were fitted using generalized least squares. Multivariate regression had to be considered due to the experiment’s nature: two different indexes were read on the same set of individuals, once at each trimester of the pregnancy. We looked for adequate global models and compared curves (as functions of time but adjusted for potential confounders) instead of comparing mean indexes between different time points. Random effects models were not used as the concern was on serial correlation and results were to be interpreted at the population level.

The response variable read for index *d* (RI or PI), in a subject with hypertensive status *h* (hypertensive or normotensive), at (continuous) time *t* was denoted by R(*d,h,t*). Dummy variables had to be considered for the categorical variables index and hypertension status; reference categories were taken to be the resistance index and the normotensive status, respectively. The fitted model was

$$\log\left(R\left(d,h,t\right)\right)=\beta_{0}\left(d,h,d\times h\right)+\beta_{1}\left(d,h,d\times h\right)\log\left(t\right)+\epsilon$$

with residuals *ϵ* following a normal distribution with mean zero and with a variance-covariance matrix that allowed for an intra-individual time autocorrelation structure of order 1 and for different variances across the indexes and the hypertensive status. Equivalently, the predicted values were given by the non-linear equation

$$R\left(d,h,t\right)=e^{\beta 0\left(d,h,d\times h\right)}t^{\beta 1\left(d,h,d\times h\right)}$$

Final regression models were chosen on the basis of the lowest BIC (Bayesian Information Criterion). Residuals normality was improved after the removal of the observations with Pearson residual greater than 3.3.

Intra-observer reliability was obtained from two readings, at the beginning and the end of the scan, on the first 60 recordings of RI and PI in the AoF. This concept concerns the degree to which measurements taken by the observer are consistent. In the present context, there was only one observer, and he was totally unaware of any of the results. Intraclass correlation coefficients (ICC) and 95% confidence intervals were calculated using a two-way mixed-effects model with absolute agreement. The reliability coefficient, which is the difference value that will be exceeded by only 5% of pairs of measurements on the same subject, was calculated as 1.96 times the standard deviation of the difference between pairs of repeated measurements.

All statistical analyses were carried out using the R language and software environment for statistical computation, version 2.12.1 [30]. The significance level was fixed at 0.05.

## Results

From January 2010 to December 2012, a total of 152 pregnant caucasian women were recruited. In the follow-up, 51 women (33.6%) were excluded because of events occurring along the pregnancy. These were gestational diabetes mellitus (n = 14), psychiatric disorders (n = 8), chronic medication beyond the established (n = 4), autoimmune disease (n = 4), later refusal to participate (n = 1), foetal pathology (n = 4), failed ultrasound evaluation at the defined schedule (n = 9) or because not all required Doppler indexes were obtained (n = 7). In particular, 8 out of 14 pregnant women who were excluded for gestational diabetes also had foetuses with growth rates above the 90th percentile. In addition, 3 in 8 pregnant women with psychiatric pathology and 4 in 9 pregnant women who missed the scans at the defined schedule, had foetuses with growth rates below the 10th percentile. Of the remaining 101 women, 71 were categorized as normotensive (NT) and 30 as chronic hypertensive (HT).

The main characteristics of women included in the study are presented in Table 1. More than half of the population had a Body Mass Index (BMI) between 18–24, and a considerable proportion of pregnant BMI greater than 25. For the variable ‘education level’, only 29% of the population attended higher education (this is justified by the fact that our hospital covers an area with important socio-economic difficulties). About half of the population were primipara (53%), none had a history of preeclampsia and only 1 reported having had hypertension during a previous pregnancy.

**Table 1:** Main characteristics of 101 women included in the study: normotensive and the hypertensive groups

		**All patients (n = 101)**	**Normotensive (n = 71)**	**Hypertensive (n = 30)**	p-value*
		**n (%)**			
Age (intervals in years)	17-24	13 (13%)	13 (18%)	0 (0%)	<0.001
	25-34	58 (57%)	46 (65%)	12 (40%)	
	35-43	30 (30%)	12 (17%)	18 (60%)	
Education level (in years)	<7	4 (4%)	3 (4%)	1 (3%)	0.616
	7-9	31 (30%)	23 (32%)	8 (27%)	
	10-12	37 (37%)	23 (32%)	14 (47%)	
	>12	29 (29%)	22 (31%)	7 (23%)	
Smoking	No	82 (81%)	60 (85%)	22 (73%)	0.301
	Yes	19 (19%)	11 (15%)	8 (27%)	
Parity	0	54 (53%)	43 (61%)	11 (37%)	0.048
	≥1	47 (47%)	28 (39%)	19 (63%)	
Body mass index at admission (Kg/m^2^)	18-24	53 (52%)	48 (68%)	5 (16%)	<0.001
	25-29	33 (33%)	16 (22%)	17 (57%)	
	30-51	15 (15%)	7 (10%)	8 (27%)	
GA at trimestral evaluation^1^	13.04 (0.68)		13.12 (0.73)	12.85 (0.50)	0.030
	20.73 (0.77)		20.77 (0.80)	20.61 (0.71)	0.327
	30.45 (1.20)		30.61 (0.75)	30.06 (1.85)	0.122
GA at delivery^1^	38.9 (1.68)		38.9 (1.67)	38.9 (1.71)	0.907

GA: gestational age; ^1^Average GA in weeks ± SD; *p - tests homogeneity of the proportions between hypertensive and normotensive; SD: standard deviation.

Regarding HT and NT groups, statistically significant differences were found for body mass index (higher BMIs in the HT group, p < 0.001) and age (increased age in the HT group, p < 0.01) – Table 1.

**Figure 2:**
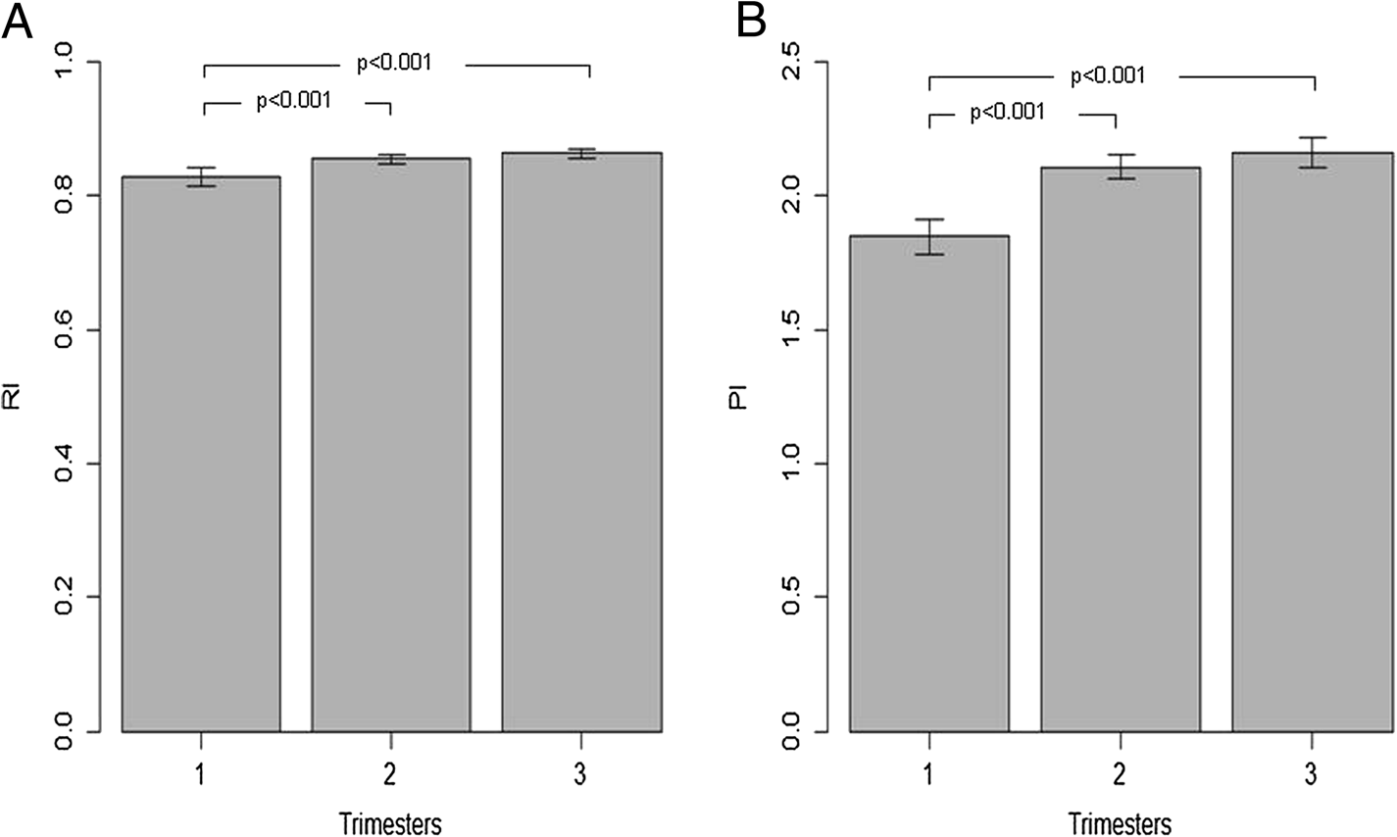
Sample means and correspondent 95% confidence intervals for foetal aortic index values during pregnancy in the all population: A- for RI (resistance index); B- for PI (pulsatility index).

The means and standard deviations of PI and RI values for AoF at every trimester and for all subjects are displayed in Table 2. The crude effect of the gestational trimester on the mean values of the AoF indexes is shown in Figure 2. There is an overall increasing trend of AoF-RI and AoF-PI mean values with time, but significant differences were only detected from the first to the second or third trimesters (p < 0.001) – Figure 2.

**Table 2:** Sample mean (and standard deviation) of foetal aortic artery indexes (RI and PI) according to the gestational trimester

	**Trimester**		
**Index**	**1**	**2**	**3**
	**All (n = 101)**		
RI	0.829 (0.068)	0.857 (0.035)	0.864 (0.037)
PI	1.850 (0.339)	2.110 (0.242)	2.163 (0.282)
	HT (n = 30)		
RI	0.811 (0.055)	0.850 (0.033)	0.864 (0.033)
PI	1.820 (0.334)	2.052 (0.211)	2.075 (0.175)
	NT (n = 71)		
RI	0.836 (0.072)	0.859 (0.036)	0.864 (0.039)
PI	1.862 (0.343)	2.134 (0.251)	2.201 (0.311)

The reliability coefficients were 0.053 and 0.153 for the pulsatility and resistance indexes respectively. The intraclass correlation coefficient for the evaluation of intraobserver reliability concerning PI (respectively RI) measurements was very high, namely 0.996 (resp. 0.936) with correspondent 95% confidence interval ranging from 0.994 to 0.998 (resp. from 0.895 to 0.961).

### Multivariate analysis and predictions

**Figure 3:**
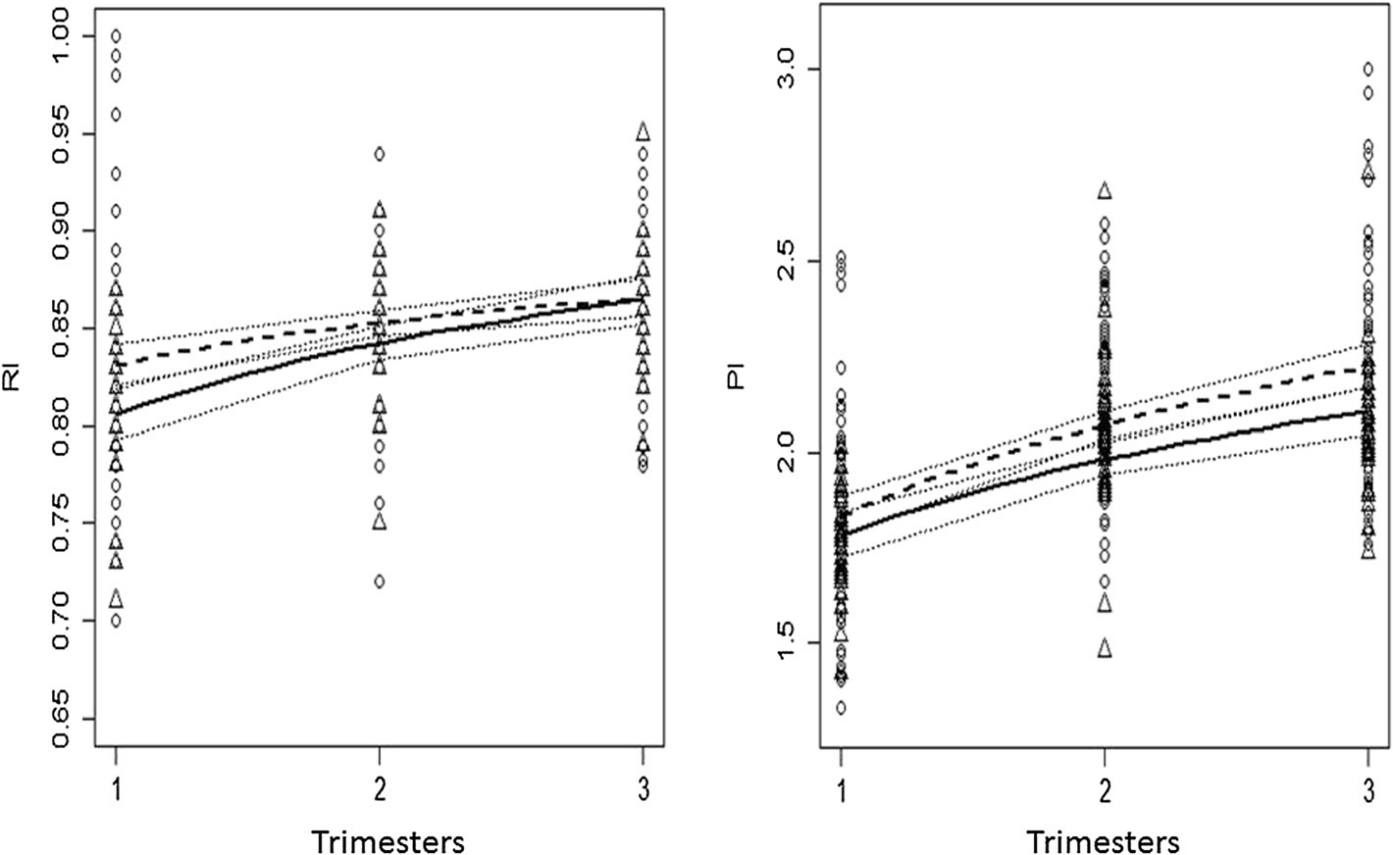
**Predicted mean resistance (RI) and pulsatility (PI) indexes of foetal aorta along pregnancy in normotensive (dashed line, circles) and hypertensive (solid line, triangles) women.** The vessel exhibits a gestational age-related increase in both indexes, but there is a noteworthy divergence of PI when groups are compared. Statistically significant differences amongst mean predicted values were found for time 2 and 3 (for time 3, is it clear from the numbers that confidence intervals just below 95% confidence level would not intersect each other). For RI and NT, CI_95%_: (0.821, 0.842), (0.846, 0.859), (0.856, 0.875) for time 1, 2 and 3, respectively; For RI and HT, CI_95%_: (0.793, 0.819), (0.834, 0.851), (0.852, 0.877) for time 1, 2 and 3, resp.; For PI and NT, CI_95%_: (1.780, 1.882), (2.035, 2.107), (2.170, 2.282) for time 1, 2 and 3, resp.; For PI and HT, CI_95%_: (1.723, 1.842), (1.940, 2.021), (2.044, 2.170) for time 1, 2 and 3, resp.

The unadjusted effect of the gestational trimesters on the mean values of the indexes is merely indicative, and multivariate analyses adjusted for potential confounders and taking the experience design into consideration had to be considered. As the difference between the average evaluation time in trimester 2 and that in trimester 1was approximately equal to the difference between the average evaluation time in trimester 3 and that in trimester 2 (more precisely, the latter is 0.95 times the former), the multivariate regression model considered the variable gestational trimester as continuous. The model also included the index and the hypertensive status as explanatory variables, as described above in the Section *Statistical Analysis*; its estimated coefficients and respective 95% confidence intervals are presented in Table 3. Known confounding variables such as age, smoking habits, body mass index and number of previous pregnancies were also taken into account in the analysis; as they were not shown to be statistically significant, they were not considered in the final model that allowed the construction/prediction of AoF-RI and AoF-PI curves – Figure 3. Log-transformations and removal of outliers were considered in order to improve the model. Seven observations (all from normotensive women and all regarding PI values, although corresponding to different time points) were identified as having Pearson residuals greater than 3.3. As its removal improved residuals normality, they were eliminated from the model fitting.

**Table 3:** Estimated coefficients and correspondent 95% confidence intervals (CI) from the regression model used to obtain the expected indexes at the different covariates combinations

**Covariates**	**Coefficient**	**95% CI**
Intercept	−0.1849*	(−0.1976, -0.1722)
log(Time)	0.0368*	(0.0199, 0.0538)
PI	0.7895*	(0.7670, 0.8120)
Hypertension	−0.0313*	(−0.0518, -0.0108)
PI × hypertension	0.0041	(−0.0307, 0.0389)
log(Time) × PI	0.1409*	(0.1109, 0.1709)
log(Time) × Hypertension	0.0276*	(0.0003, 0.0550)
log(Time) × PI × Hypertension	−0.0528*	(−0.0992, -0.0064)

*significant coefficient for a significance level of 0.05.

At baseline, in the first trimester and for RI only, a significant difference between its mean values in hypertensive and normotensive women was identified - Table 3. However, as gestational time progressed, that effect ceased its significance and the obtained mean values for hypertensive and normotensive women were no longer significantly different from each other. On the other hand, for PI, the scenario was the opposite: although no significant differences between hypertensive and normotensive women were detected at baseline, the mean values of the two groups at the second and third trimesters differed significantly from each other (Table 3).

The model equation written in the original variables had multiplicative effects, implying that coefficients estimates had to be interpreted accordingly. For example, for a fixed index, a fixed hypertensive status and a fixed time point *t*, the response value obtained for time *kt* is *k*^*β*1^ times the response value obtained for *t*, for any real number *k*. Nonetheless, for any fixed index and hypertensive status, a significant effect of time was observed. All curves obtained in Figure 3 are significantly different from a flat line therefore reflecting an increase in RI and PI values along the gestation. This follows directly from the statistical significance of all terms containing log(Time) in the considered model (Table 3).

## Discussion

Chronic hypertension is a relatively common disorder that affects 1–5% of pregnant women, depending on the population studied and the diagnostic criteria employed [31]. Because of increasing worldwide maternal age, obesity and type 2 diabetes, it is expected that the prevalence of chronic hypertension in pregnancy will continue to increase [32] and be accompanied by known complications [19,20].

The application of Doppler sonography in the analysis of foetal blood flow velocities to assess fetoplacental circulation is now part of the management of high-risk pregnancies [1,3,33]. In the current study, reliability evaluation demonstrated that a Doppler blood flow measurement of the PI-UtA and RI-UtA was highly repeatable within our sonographer [3,18]. We used ICC to assess repeatability since there is sufficient consensus in the scientific literature to consider that values for ICC > 0.7 reflect very low measurement error [34,35].

It has been questioned whether disturbance of uterine vessel haemodynamics, determinant for foetal nourishment, may result in impedance derangement at the foetal circulation. In fact, increased peripheral vascular resistance in women with chronic arterial hypertension is mediated by some agents that cross the placenta and, upon the activation of specific receptors in the foetal vessel walls, can induce local circulatory changes [36,37]. Likely targets include the foetal aorta as its Doppler frequency spectrum is modulated by a variety of circulatory variables as foetal cardiac output, arterial impedance of the peripheral vascular system and blood viscosity [14]. Indeed, the study of the foetal aorta was credited with additional advantage for the diagnostic [9,12,13] or prognostic [2,10,14] evaluation of conditions associated with enhanced foetal risk.

The flow profile in the foetal proximal descending aorta is characterized by a steep systolic rise with a postsystolic notch as well as by relatively low end-diastolic forward flow velocities [3]. In our study, no gross morphological differences were identified in the typical fluxometric spectra of the foetal proximal descending aorta between normal and hypertensive groups of patients along all trimesters. However, an increase of AoF-RI and Aof-PI with advancing gestation was noticed, in accordance with previous studies in the second and third trimesters of uneventful pregnancies [3,18]. This observation is likely to result from increased myocardial contractility and decreased middle cerebral arteries impedance; these events favour diastolic forward flow decrease in AoF, and enhanced AoF-PI and AoF-RI. The novelty of the present study is the longitudinal assessment of such indexes, starting at the first trimester of pregnancy. Moreover, instead of studying adverse outcomes in a group with a specific pregnancy disorder the authors examined the effect of different circulatory conditions, normotensive and hypertensive, having in common the absence of adverse maternal-foetal and perinatal outcomes.

The aorta of foetuses from pregnant women with chronic arterial hypertension presented a haemodynamic pattern similar to that from healthy mothers. In fact, a crude significant increase in the average value of RI and of AoF-PI from the first to the second trimester and from the first to the third trimester was found in both groups. However, the global model showed that while RI trends were converging to the same value as time progressed, the PI values exhibited a divergent trend. More precisely, while starting at not significantly different mean values, the two groups then evolved to significantly different values in the second and third trimesters.

These findings are quite interesting, particularly the regular, progressive divergence of PI, a most valued impedance index that appears to describe the shape of the velocity waveform much better [17,38]. Such regular trend suggests that an adaptive, counter-resistant compensatory mechanism is continuously activated at the foetal aorta to compensate uncertain requirements that pregnant women with chronic arterial hypertension, albeit stable, cannot provide to the growing foetus. We hypothesize that in hypertension, the placental barrier is crossed by a vasoactive compound or compounds to which the foetal aorta responds by blunting the increasing impedance observed in normal pregnancy. However, we believe that this difference should not be attributed only to the aortic artery. Thus, additional studies are needed to explore differences in output and contractility of the foetal heart, as well as the vascular compliance, blood viscosity and impedance of the arterial vascular system, of healthy and chronic hypertensive pregnant women.

### Study limitations

(1) The study was conducted in a sample of uneventful pregnancies. Therefore, it is not possible to establish causal relationships to the development of severe disorders or appearance of serious foetal events that future studies with specific risk groups may provide. (2) Blood pressure was not measured at the moment of each Doppler study; (3) Starting at the first trimester, HT patients were medicated daily with low-dose acetylsalicylic acid, whose contribution can’t be formally excluded.

## Conclusions

In pregnancies with normal outcomes, maternal stable hypertension is associated with a progressive increase in foetal descending aorta PI albeit at a less pronounced rate, when compared to the normotensive condition. The data suggest an adaptive mechanism imparted by the pregnancy condition and pave the way for an approach in unfavorable pregnancy conditions, which will help to understand the still elusive foetal-placental bed circulation interaction.

## Abbreviations

AoF: Foetal proximal descending aorta; BMI: Body mass índex; HT: Hypertensive; GA: Gestational age; NT: Normotensive; PI: Pulsatility index; RI: Resistance index; ICC: Intraclass correlation coefficient.

## Competing interests

The authors declare that they have no competing interests.

## Authors’ contributions

LG-M and HA designed the study, analyzed the data and wrote the manuscript; AC coordinated quality control of ultrasound data; JS coordinated review of clinical cases and organization of study groups; RG performed all statistical analyses; ASC contributed to the critical revision of the manuscript; FM designed the study. All authors contributed to the data interpretation and the final version of the manuscript, which they all approve.
